# Respiratory Syncytial Virus–related Community Chronic Obstructive Pulmonary Disease Exacerbations and Novel Diagnostics: A Binational Prospective Cohort Study

**DOI:** 10.1164/rccm.202308-1320OC

**Published:** 2024-03-19

**Authors:** Dexter J. Wiseman, Ryan S. Thwaites, Andrew I. Ritchie, Lydia Finney, Mairi Macleod, Faisal Kamal, Hassan Shahbakhti, Lisa H. van Smoorenburg, Hiub A. M. Kerstjens, Joanne Wildenbeest, Deniz Öner, Jeroen Aerssens, Guy Berbers, Rutger Schepp, Ashley Uruchurtu, Benedikt Ditz, Louis Bont, James P. Allinson, Maarten van den Berge, Gavin C. Donaldson, Peter J. M. Openshaw, Jadwiga Wedzicha, on behalf of RESCEU Investigators, Harish Nair

**Affiliations:** ^1^ National Heart and Lung Institute, Imperial College London, London, United Kingdom;; ^2^ Chelsea and Westminster National Health Service Foundation Trust, London, United Kingdom;; ^3^ Research and Early Clinical Development, Respiratory and Immunology, Biopharmaceuticals R&D, AstraZeneca, Cambridge, United Kingdom;; ^4^ Department of Pulmonology and Groningen Research Institute for Asthma and COPD, University of Groningen, University Medical Center Groningen, Groningen, the Netherlands;; ^5^ Department of Paediatric Infectious Diseases and Immunology, Wilhelmina Children’s Hospital, University Medical Centre Utrecht, Utrecht, the Netherlands;; ^6^ Centre of Infectious Disease Control, National Institute of Public Health and the Environment, Bilthoven, the Netherlands; and; ^7^ Infectious Diseases Translational Biomarkers, Janssen Pharmaceutica, Beerse, Belgium

**Keywords:** respiratory syncytial virus, chronic obstructive pulmonary disease, rhinovirus

## Abstract

**Rationale:**

Respiratory syncytial virus (RSV) is a common global respiratory virus that is increasingly recognized as a major pathogen in frail older adults and as a cause of chronic obstructive pulmonary disease (COPD) exacerbations. There is no single test for RSV in adults that has acceptable diagnostic accuracy. Trials of RSV vaccines have recently shown excellent safety and efficacy against RSV in older adults; defining the frequency of RSV-related community infections and COPD exacerbations is important for vaccine deployment decisions.

**Objectives:**

This prospective study aimed to establish the frequency of outpatient-managed RSV-related exacerbations of COPD in two well-characterized patient cohorts using a combination of diagnostic methods.

**Methods:**

Participants were recruited at specialist clinics in London, United Kingdom, and Groningen, the Netherlands, beginning in 2017 and observed for three consecutive RSV seasons, during exacerbations, and at least twice yearly. RSV infections were detected by RT-PCR and serologic testing.

**Measurements and Main Results:**

A total of 377 patients with COPD attended 1,999 clinic visits and reported 310 exacerbations. There were 27 RSV-related exacerbations (8.7% of the total); of these, seven were detected only by PCR, 16 only by serology, and four by both methods. Increases in RSV-specific Nucleoprotein antibody were as sensitive as those in the antibody to Pre-Fusion or Post-Fusion for serodiagnosis of RSV-related exacerbations.

**Conclusions:**

RSV is associated with 8.7% of outpatient-managed COPD exacerbations in this study. Antibodies to RSV Nucleoprotein may have diagnostic value and are potentially important in a vaccinated population. The introduction of vaccines that prevent RSV is expected to benefit patients with COPD.

**At a Glance Commentary**

**Current Scientific Knowledge on the Subject**

Our knowledge regarding the burden of respiratory syncytial virus (RSV) among older adults and those with comorbidities is growing as a result of greater awareness of the virus and improved diagnostic techniques. Although there have been studies investigating the burden of RSV among inpatient chronic obstructive pulmonary disease (COPD) cohorts, to date, there has not been a large study evaluating the burden of RSV in the outpatient COPD setting.

**What This Study Adds to the Field**

This study adds to the field by defining the burden of RSV among an outpatient COPD group, including defining the severity of RSV exacerbations versus those caused by other pathogens. This is important information for decision makers and physicians with regard to the novel, effective adult RSV vaccines.

Chronic obstructive pulmonary disease (COPD) inflicts a significant burden on healthcare systems throughout the world (1). In high-income settings, smoking remains the commonest cause (2), but high rates in developing countries are attributable to poor quality of indoor and outdoor air in addition to tobacco smoke (3). The global prevalence of COPD among people aged 30–79 years has been estimated at 10.3% (3).

Acute exacerbations of COPD are responsible for approximately 2.4% of all acute hospitalizations in England, with a median length of stay of 7 days (4, 5). COPD exacerbations requiring hospital admission are associated with an 11.6% in-hospital mortality rate (6). For those readmitted after hospital discharge, the mortality rate increases to 37% (6). Although approximately half of all exacerbations are not reported to healthcare professionals (7), those unreported exacerbations are associated with more rapid progression of disease and accelerated decline in lung function (as measured by FEV_1_) (8). Patients with frequent exacerbations have poorer quality of life (9). Thus, preventing exacerbations is a crucially important strategy toward slowing COPD progression and improving the lives of patients.

Current virally related exacerbation prevention strategies include annual vaccination against influenza (10). However, other viruses, such as respiratory syncytial virus (RSV), trigger exacerbations, causing substantial morbidity and mortality. The all-cause mortality rate for RSV-related admissions in adults is greater than that for influenza-related admissions (18.4% vs. 6.7%) (11). RSV is also associated with longer hospital stays, a higher risk of pneumonia, and ICU admissions (12). However, although the prevalence of RSV among patients with COPD in the inpatient setting has been explored (13–15), community RSV infections are likely often missed, leaving their impact poorly defined (16).

Diagnosing RSV in an adult of any age is not simple. Molecular and serologic methods are now the mainstay of diagnosis (17), but PCR-based diagnostics are not 100% sensitive. An increase in RSV-specific serum antibody is an alternative indicator of an RSV infection (17), but allows only retrospective diagnosis.

Delineating how RSV contributes to COPD exacerbations is especially important in predicting the potential impact of recently available vaccines that are highly effective in preventing RSV lower respiratory tract disease in older adults (18–20). Targeting of vaccines and antiviral therapy to those at the greatest risk will be important in obtaining maximal benefit from these new measures. To address this issue, we prospectively recruited patients with COPD at specialist respiratory clinics in London, United Kingdom, and Groningen, the Netherlands, to investigate the role of RSV in causing exacerbations in the outpatient setting. RSV exacerbation severity was also investigated among the London cohort.

The newly approved older adult vaccines all incorporate viral F (Fusion) Protein (19, 20) and induce F Protein–specific antibodies. We therefore aimed to determine the value of antibodies against viral Nucleoprotein (N protein) using a pentaplex serological assay developed by the Dutch National Institute for Public Health and the Environment (RIVM) (21) in a subanalysis of samples from the London cohort. Using serological assays to a non-F antigen could be useful for identifying RSV infections in vaccinated cohorts.

Some of the results of these studies have been previously reported in the form of a preprint (*The Lancet*, June 23, 2023; https://papers.ssrn.com/sol3/papers.cfm?abstract_id=4487116).

## Methods

### Ethics Statement

All relevant research ethics committee (REC) and ethics permissions were obtained before the start of this project. The U.K. arm of this study was approved by the Harrow REC (reference 17/LO/1424; date of REC approval, October 18, 2017), whereas the Netherlands arm was approved by the local medical ethics committee (University of Groningen; METc 2017/015). All participants gave informed written consent.

### Inclusion and Exclusion Criteria

We included patients with spirometry evidence of airflow obstruction (FEV_1_/FVC ratio ⩽0.7) aged ⩾40 years who had accrued a ⩾10–pack-year smoking history (current or former smokers) and committed to attending research visits to the clinic. Participants were excluded if they also had a diagnosis of asthma, significant bronchiectasis, or any other significant respiratory disease (including lung cancer). Immunosuppressed patients and those receiving long-term steroid therapy (⩾10 mg/d prednisolone) were also excluded.

### Study Design

Participants were clinically reviewed at intervals of 3–6 months and completed spirometry, blood tests, sputum collection (if expectorating), clinical observations, a COPD Assessment Test (CAT), and sputum score. Social questionnaires were also completed annually (Table E3 in the online supplement).

Participants were instructed to contact the study site through a dedicated phone number within 48 hours of the onset of an exacerbation. At exacerbations, participants were brought into the study center for clinical assessment and sampling (including a nasopharyngeal [NP] swab). During these visits, participants received a CAT score and a sputum score and answered exposure questions.

**Figure 1.**
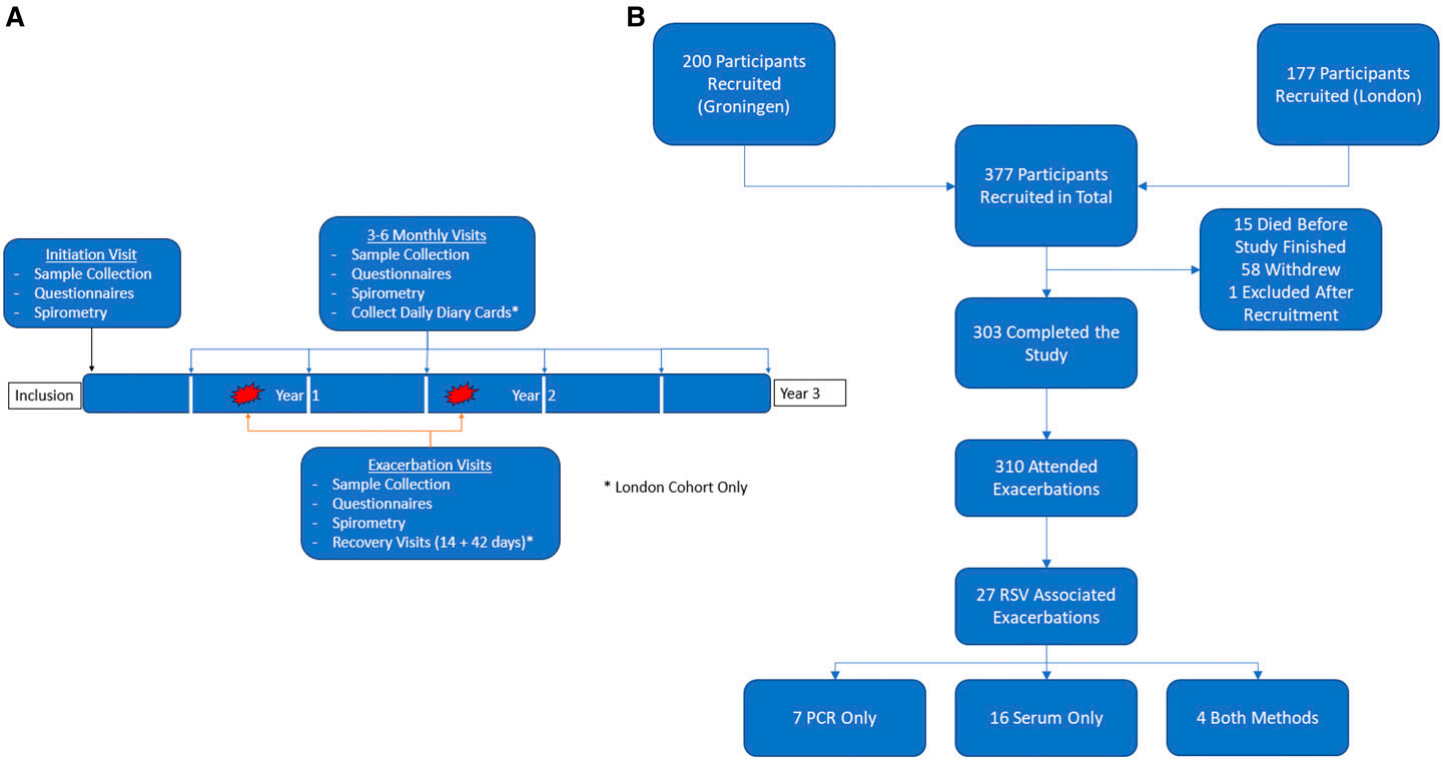
RESCEU (Respiratory Syncytial Virus Consortium in Europe) chronic obstructive pulmonary disease cohort study design and recruitment. (*A*) Study design at the London and Groningen recruitment sites. Some additional analyses were conducted at the London site (asterisks). (*B*) Consolidated Standards of Reporting Trials diagram for participant recruitment, retention, and observation of chronic obstructive pulmonary disease exacerbations. RSV = respiratory syncytial virus.

Exclusively at the London site, participants completed daily diary cards, recording changes in symptoms, treatment, and daily peak flow rates. Participants at the London site were seen again at 2 weeks and 6 weeks after the onset of an exacerbation to assess recovery and collect serum (this was already established practice within this research cohort) (Figure 1A).

### Defining and Treating Exacerbations

Exacerbations were defined by two new symptoms (at least one major) for two or more consecutive days (7). Major symptoms were increased breathlessness, increased sputum volume, or intensified sputum color (7). Minor symptoms were sore throat, fever, worsening cough, increased wheeze or chest tightness, and subjective assessment of having an upper respiratory tract infection (7). Exacerbations were defined as ending after two consecutive symptom-free days, the last day of symptoms being considered the end date. If another exacerbation started within 5 days of the end of the exacerbation, this was counted as a prolongation of the original exacerbation (7) (*see* Table E1 for definitions of the causes of exacerbations). Exacerbations were treated per local guidelines and were classified as “attended” if the participant attended the study site per protocol. Exacerbations were defined as “unattended” if the participant did not attend for any reason and the exacerbation was later diagnosed retrospectively from the diary card data (London cohort only).

### Molecular (PCR) Viral Testing

NP swabs and, when a sample was obtained, sputum were analyzed using multiplex quantitative RT-PCR by GlaxoSmithKline, with the samples tested for RSV A, RSV B, rhinovirus, influenza (i.e., flu) A, flu B, human coronavirus (HCV)-NL63, HCV-229E, HCV-OC43, human bocavirus, human enterovirus, human adenovirus, parainfluenza virus (PIV)-1, PIV-2, PIV-3, PIV-4, and human metapneumovirus.

### Serological RSV Testing

Serum Pre-F and Post-F RSV protein–specific IgG were analyzed by Janssen using an internally validated ELISA (22). A fourfold titer increase between a pre- and postexacerbation sample pair was considered the threshold for serological detection of an RSV-associated exacerbation as previously described (17).

Exclusively in the London cohort, a pentaplex serology assay (generated by the RIVM as part of the RESCEU [Respiratory Syncytial Virus Consortium in Europe]; https://resc-eu.org/) was used to test for a wider array of RSV antigen–specific IgGs (21). This assay was run using samples from only the London cohort as a result of limited reagent availability. This testing evaluated concentrations of IgG antibodies specific to RSV Pre-F protein, Post-F protein, N protein, Glycoprotein (G) a, and Gb antigens. A fourfold change in any of the five antibody types tested was considered a positive result. Multiplex immunoassay plates were analyzed on a Bio-Plex 200 plate reader, with settings of High RP1 Target, 50 beads per region, and a 60-second timeout. Samples for the generation of standard curves were provided by Guy Berbers (RIVM) for this assay. All exacerbations tested were sampled during (or before) and after the exacerbation (<90 days after the exacerbation), with a maximum of 180 days between samples per published literature (23).

### Statistical Analysis

All statistical analysis were performed using STATA 16 and GraphPad Prism V9. Inspection of histograms demonstrated that most data were not normally distributed, and nonparametric statistical tests were employed throughout. Mann-Whitney tests were used for comparisons of two groups, and Kruskal-Wallis tests with Dunn’s multiple comparisons tests were used for comparisons of more than two groups.

In the London cohort subsample, time to recovery was calculated using participant diary cards and by counting symptomatic days (7). When the participant had two symptom-free days and had not experienced another exacerbation by diary card criteria within 5 days, they were considered recovered. Participants who experienced another exacerbation, by diary card criteria, before the postexacerbation follow-up visit were excluded from serological analysis because the changes could not be definitively linked to a specific exacerbation. Change in CAT score was calculated as the CAT score at the exacerbation visit minus the total CAT score at the previous baseline visit (provided it was >5 weeks since the end of the previous exacerbation and >2 weeks before the exacerbation visit).

## Results

### Recruitment and Demographic Data

Between November 13, 2017, and December 13, 2019, we recruited 377 patients: 177 at Imperial College London and 200 at University Medical Center Groningen (Figure 1B) (*see* Figure E5 for detailed recruitment results). Demographic characteristics were similar between sites, but participants in the London cohort were older (*P* < 0.005) and more likely to be current smokers (*P* = 0.005) (Table 1).

**Table 1.** Comparison of Chronic Obstructive Pulmonary Disease Cohort Demographic Characteristics between the London and Groningen Sites

Characteristic	London (*n* = 177)	Groningen (*n* = 200)	*P* Value
Age, yr	72.11 (71.0–73.2)	67.6 (66.6–68.5)	<0.005*
Body mass index, kg/m^2^	27.03 (26.30–27.77)	27.04 (26.19–27.87)	0.991
FEV_1_ % predicted	65.2%	64.3%	0.265
Gold stage 3/4 on spirometry	25%	23%	0.425
FEV_1_/FVC ratio	55.4% (46.9–62.3)	52.5% (41.2–62.7)	0.274
Pack-years of smoking	50.8 (46.2–55.4)	45.6 (41.1–50.1)	0.117
Current smoker	60 (34%)	42 (21%)	0.005*
Male sex	112 (63%)	138 (69%)	0.597

Data presented as median (IQR) where applicable.

* Significant at *P* < 0.05.

There were 310 attended exacerbation events in the cohort (188 in London; 122 in Groningen). A total of 303 of 377 participants (80.37%) completed the study from recruitment until the end date: 155 in London and 148 in Groningen. Of those who did not complete the study, 15 participants died before the end date (3.98%; eight in London and seven in Groningen), 58 withdrew before the study end date because of disease or personal circumstances (15.38%; 14 in London and 44 in Groningen), and one (0.27%) was excluded after enrollment in Groningen (Figure 1B). Median follow-up durations per participant were 748 days (IQR, 424–814 d) in the London cohort and 1,132 days (IQR, 948–1,274 d) in the Groningen cohort. The total follow-up time was 108,557 patient-days, or 296.8 patient-years. The London cohort also had 248 documented unattended exacerbation events per diary card criteria.

### Frequency of RSV-associated Exacerbations

We first sought to identify RSV cases using molecular testing of airway samples.

#### NP PCR

NP swabs were collected at 297 of 310 attended exacerbations (96%) and analyzed by multiplex quantitative RT-PCR. A total of 206 sputum samples were analyzed, including 104 from exacerbation visits, 20 at follow-up, and 82 from baseline visits.

Six of 297 NP swabs (2.02%) were positive for RSV (five RSV B, one RSV A). Viral load ranged from 6,853 to 86,503,569 copies/ml, with a mean of 15,924,109 copies/ml.

#### Sputum PCR

From the 104 exacerbation sputum samples, eight (7.69%) were positive for RSV (three RSV B, five RSV A). Viral load ranged from 691 to 142,438,576 copies/ml, with a mean of 35,632,817 copies/ml.

There was a higher positivity rate for RSV from sputum compared with NP swabs (*P* = 0.0061). None of the baseline sputum samples were positive for RSV. Three RSV-related exacerbations were identified by PCR from sputum and NP swabs.

#### Serology

We next sought to serologically identify RSV cases missed by molecular testing. In total, 16 RSV-associated exacerbations were identified exclusively using the Janssen serology assay.

Seven RSV-associated exacerbations were identified exclusively using quantitative PCR, and four were positive by both methods. This resulted in a total of 27 RSV-related exacerbations using this combined testing approach, therefore associating RSV with 8.7% (27 of 310) of outpatient-managed COPD exacerbations (Figure 1B).

There was a greater variety of viruses present from the London samples, with 14 different viral pathogens present in the exacerbation NP samples and 14 in the exacerbation sputum samples. In contrast, there were only four different viral pathogens present in the Groningen NP exacerbation samples and three in the Groningen sputum samples (Figures E1 and E2).

### Secondary Evaluation in the London Cohort

Further diagnostics were available for the London cohort in the form of a pentaplex serological assay (21). We also had access to bacteriology (*see* Figure E3) as well as markers of severity from the London cohort to allow for more detailed exacerbation severity analysis.

We first sought to assess the severity of the attended exacerbations experienced by the London cohort and distinguish differences based on the exacerbation-associated pathogens. FEV_1_ change at exacerbation, change in CAT score and time to recovery, fibrinogen, C-reactive protein (CRP), and white blood cell count for all the causes of exacerbation were considered (Table 2).

**Table 2.** Exacerbation Severity Measures by Exacerbation Cause

	RSV (*n* = 16)	Rhinovirus (*n* = 30)	Flu (*n* = 9)	Other Viruses (*n* = 16)	Bacterial (*n* = 24)	Viral + Bacterial (*n* = 15)	Unknown (*n* = 64)
Days to recovery	6.5 (3.5–12.5)	20 (11.5–30.5)*	18 (8–31)*	13 (7–29)*	15 (6–26)*	15.5 (10–36)*	11 (5–25)*
Fibrinogen, g/L	4 (3.6–4.9)	4.2 (3.5–4.7)	3.9 (3.4–4.4)	4.2 (3.4–5.3)	3.8 (3.4–4.4)	4.2 (2.7–5.8)	3.8 (3–4.5)
CRP, mg/L	9 (3–47)	10 (6–21)	24 (12–41)	7 (3–14)	8 (4–20)	24 (2–64)	6 (2–13)
WBC count, ×10^9^/L	8.2 (7.5–9.7)	8.3 (6.9–9.1)	8.5 (7–10.1)	6.5 (5.7–9.9)	9.4 (7.7–10.9)	10.5 (8.5–12.4)	8 (6.9–9.6)
FEV_1_% change	−5 (−1 to −12)	−6 (−1 to −12)	−2.9 (−1 to −13)	−12.5 (−2 to −18)	−8.9 (1 to −14)	−4 (−2 to −8)	−8.9 (−2 to −20)
CAT score change	8 (2 to 13)	4 (0 to 8.5)	9 (5 to 12)	0.5 (−7 to 8)*	4 (1.5 to 7)	6 (−1 to 8)	4 (0 to 9)

*Definition of abbreviations*: CAT = COPD Assessment Test; CRP = C-reactive protein; RSV = respiratory syncytial virus; WBC = white blood cell.

Data presented as median (IQR). Kruskal-Wallis and Dunn’s posttest were used to test significance.

* Significant at *P* < 0.05 vs. RSV-related exacerbations.

**Figure 2.**
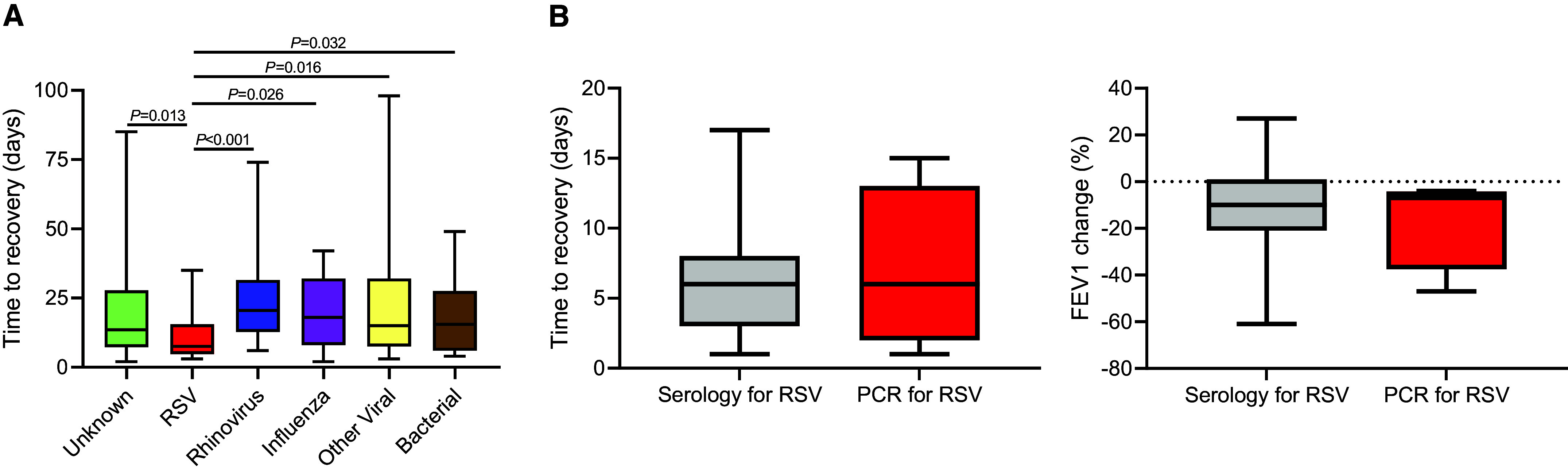
(*A*) Respiratory syncytial virus (RSV) exacerbations are shorter than those caused by other pathogens. Time to recovery was assessed for all exacerbations, and durations were compared between exacerbation-associated pathogens (unknown, *n*  = 64; RSV, *n* = 16; rhinovirus, *n* = 30; influenza, *n* = 9; other virus, *n* = 16; bacteria, *n* = 24). Symptom duration was calculated from diary cards on which the data were complete. (*B*) Exacerbation severity is not related to diagnostic method. Time to recovery (in days) in RSV-associated exacerbations by diagnostic method (*P* = 0.947, Mann-Whitney *U* test; serology diagnosis, *n* = 11; PCR diagnosis, *n* = 5). FEV_1_% change in RSV-associated exacerbations by diagnostic method (*P* = 0.947, Mann-Whitney *U* test; serology diagnosis, *n* = 11; PCR diagnosis, *n* = 5). Data in *A* were analyzed using a Kruskal-Wallis test with Dunn’s *post hoc* test, with *P* values denoting comparisons between RSV and all other exacerbation etiologies.

Investigating the duration of illness, there was a statistically significant difference between exacerbation-associated pathogens (Kruskal-Wallis ANOVA, *P* = 0.026; Figure 2A) and RSV exacerbations that were shorter in duration. Indeed, after accounting for multiple testing using a Dunn’s test, RSV-associated exacerbations were significantly shorter than exacerbations with all other causes (all *P* < 0.05; Table E2).

We next considered other metrics of exacerbation severity. There was no difference in FEV_1_% change (Figure E4), CAT score, or CRP at exacerbations between the different pathogens based on a Kruskal-Wallis test with a Dunn’s post-test. There was also no statistically significant difference when analyzing fibrinogen, CRP, or white blood cell count (Table 2). There was no difference in exacerbation severity across any of the metrics evaluated when comparing RSV diagnostic methods (PCR and serology; Figure 2B). Also, no correlation was found between viral load and exacerbation severity.

Analyzing the subjective symptoms reported by participants with different causes of COPD exacerbations in this study, there was no observable difference in specific symptoms from diary cards or CAT questionnaires between any pathogens (data not shown).

We next sought to determine the value of antibodies against RSV-specific N protein as a method of diagnosing RSV using a recently described pentaplex serology assay (21). Because of reagent availability, this substudy was conducted in only the London cohort samples.

To evaluate the pentaplex assay, first, PCR-confirmed RSV-associated exacerbations (*n* = 5) were compared with exacerbations associated with rhinovirus (*n* = 14), the single most common agent identified.

**Figure 3.**
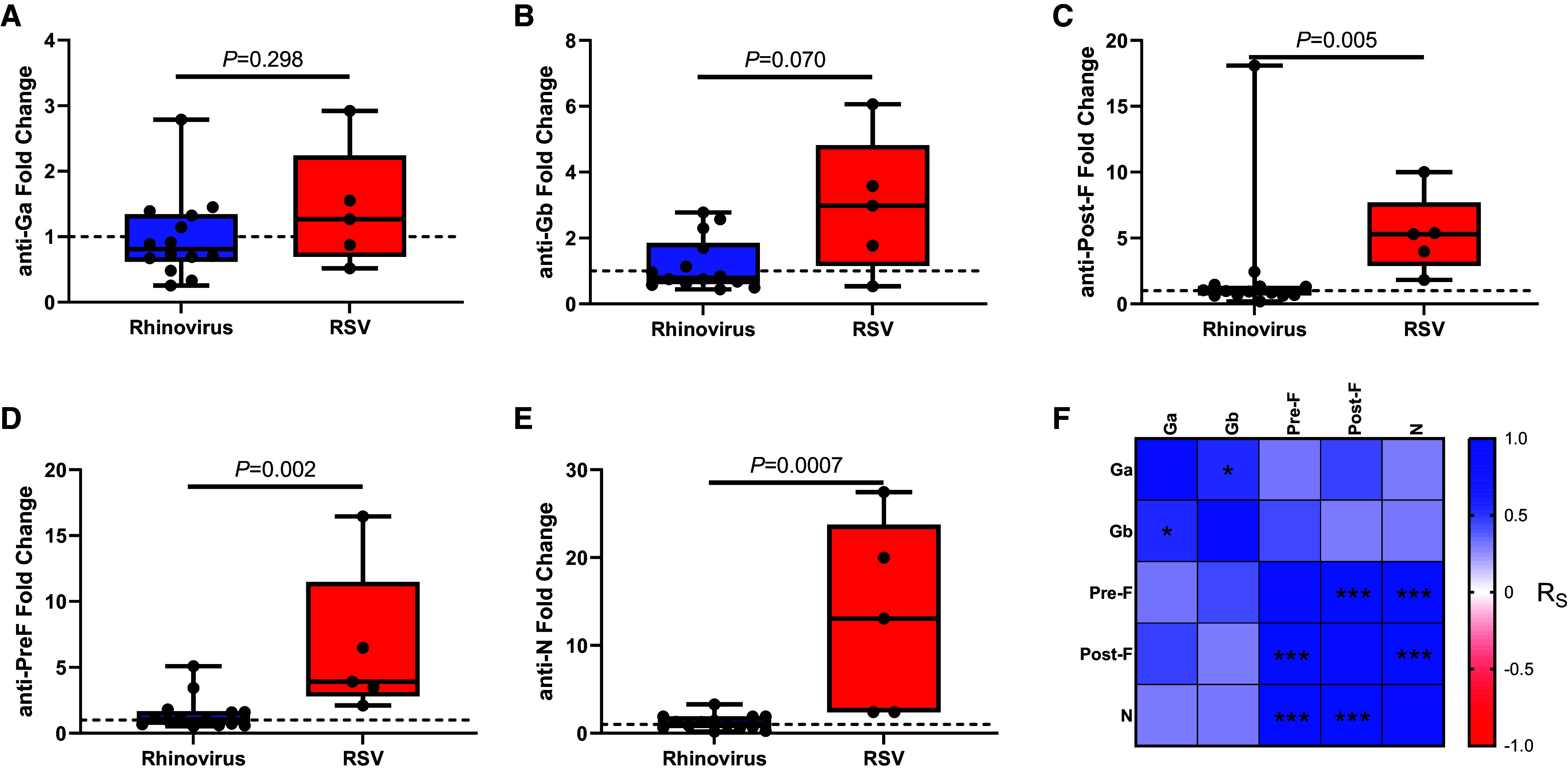
Respiratory syncytial virus (RSV) exacerbations are associated with serological responses to diverse RSV proteins. Fold change in plasma IgG titer to RSV proteins (*A*) Glycoprotein a, (*B*) Glycoprotein b, (*C*) Post-Fusion, (*D*) Pre-Fusion, and (*E*) Nucleoprotein in RSV- and rhinovirus-associated exacerbations. (*F*) Correlation matrix of fold changes (before vs. after exacerbation) between antibody specificities. Significance was tested in *A–E* using Mann-Whitney *U* tests between groups, with the boxes representing medians and IQRs and whiskers representing ranges. (*F*) Spearman correlation values between paired data (*P < 0.05 and ***P < 0.001). F = Fusion; G = Glycoprotein; N = Nucleoprotein

There was no statistically significant difference in fold change for Ga or Gb antibodies when comparing rhinovirus exacerbations versus RSV exacerbations, likely because of the mixture of RSV-A and RSV-B infections in this cohort and the low numbers of RSV PCR-positive cases for comparison (Figures 3A and 3B). However, there was a statistically significant increase in Pre-F, Post-F, and N protein titers after an RSV exacerbation compared with a rhinovirus exacerbation (Mann-Whitney *U* test, *P* = 0.002, *P* = 0.005, and *P* = 0.006, Figures 3C–3E, respectively).

To validate the pentaplex results, the correlation between the Pre-F and Post-F pentaplex data and the ELISA data was analyzed. Initially, we compared cases in which samples were analyzed using both methods (*n* = 274 pairs). Of those pairs, 166 had pre- and postexacerbation samples, enabling comparison of seroconversion events between methods.

Pre-F and Post-F antibodies analyzed using the two different methods agreed with each other by reaching or not reaching a fourfold increase on 153 of 166 readings (92.2%). A Cohen’s κ agreement was used to evaluate this similarity, and the agreement was significantly greater than the 85.77% expected agreement (κ = 0.450; SE = 0.065; *P* < 0.001).

These statistics were calculated with the fourfold cutoff value as positive or negative (i.e., binary). In agreement with this, close correlations were seen between continuous log-transformed titer data when all 275 matched data points were compared for Pre-F (*R* = 0.558, *P* < 0.0001) and Post-F (*R* = 0.517, *P* < 0.0001) proteins (Figure E6 in the online supplement). These data indicate that multiplex immunoassays provide similar performance to single-antigen assays for identifying seroconversion events, while adding that non-F antigens may be valuable for profiling infections in the postvaccine era.

Together, these data demonstrate that RSV accounted for approximately 8.7% of COPD exacerbations across two large European cohorts. In the London cohort, RSV-associated COPD exacerbations were associated with similar objective and subjective measures of severity relative to other causes of exacerbations. However, RSV exacerbations tended to resolve more quickly.

## Discussion

We found that RSV infection was associated with 8.7% of exacerbations in our cohorts but confirmed that many infections would be missed with a reliance on PCR alone. RSV infection was identified as the cause of 27 of 310 attended exacerbations. Of these, 7 were detected by PCR alone and 16 were detected exclusively by serology; 4 were detected by both methods. Therefore, 59% of RSV-associated exacerbations were negative on PCR despite a swab being obtained by highly trained staff within 5 days of symptom onset.

As of February 2024, there were three RSV vaccine candidates in phase II trials and one in phase III trials aimed at older adults, with two approved for marketing in the United States. Each of these vaccines seem highly effective (19, 24). Because we and others have shown that RSV infection is a significant cause of exacerbations of COPD, such patients should be considered a priority for future studies of the effect of vaccination on exacerbations and lung function decline. Given the apparent inefficiency of PCR in the detection of RSV disease in adults, it is possible that vaccination will have a considerably greater effect than that estimated from studies that used PCR alone.

The pentaplex serology assay (used in the London cohort) that specifically tested for the N, Pre-F, and Post-F antibodies was as sensitive as the ELISA method for diagnosing RSV. The N protein antibody has not previously been used to detect and diagnose RSV in older adults or patients with COPD. Titer increases for N protein–specific antibodies appear to be greater than those for Pre-F– and Post-F–specific antibodies when an RSV-associated exacerbation has occurred (Figure 3). N protein serological testing could enhance our ability to diagnose RSV infections. This may be of particular importance in vaccinated cohorts, as most vaccines in development target the F protein alone (19, 20), with an N protein–specific antibody test potentially offering a tool to serologically define breakthrough infections after vaccination.

In agreement with the existing literature, rhinovirus and RSV were the two viruses most commonly associated with COPD exacerbations (25). We found that RSV-positive exacerbations were shorter in duration than RSV-negative exacerbations. To our knowledge, this is the first time that the duration of RSV-related exacerbations has been investigated relative to other causes of COPD exacerbations in the outpatient setting. The possible reasons for RSV-associated exacerbations being shorter include delayed diagnosis because early RSV symptoms in patients with COPD may be missed relative to the symptoms that occur when exacerbations are caused by other triggers. Although evidence regarding RSV persistence in COPD (in which the virus lies dormant in the airways) is conflicting (26, 27), some patients being primed to clear infections could offer an alternative explanation.

There is a propensity for RSV to replicate in the lower airways, so nasal swabbing alone might not be sufficient to obtain a diagnosis (28), and lower airway sampling should also be considered. However, it was not feasible to perform bronchoscopy at the time of exacerbations in the participants with COPD in this study. The next best method for sampling the lower airways is to analyze sputum, which also enables sputum culture to identify concomitant bacteria or bacterial causes of exacerbation. No RSV was detected in the baseline sputum samples during this study, but there was a higher positivity rate for RSV in sputum across both study sites: 8 of the 104 exacerbation sputum samples were positive for RSV compared with 6 of the 297 exacerbation NP swabs. It would be valuable to perform induced sputum cultures in future studies to see if this enhances RSV detection, but sputum induction can be poorly tolerated (29). Using a combination of viral PCR of NP swabs and lower airway samples alongside serological methods should increase the rate of RSV diagnosis in future studies.

The lack of variety in viral pathogens from the Groningen samples (sputum and NP swabs) stands out. London is a large city with a large migrant population of international travelers and tourists, perhaps exposing Londoners to a greater variety of exacerbation-causing pathogens. There is some evidence of greater viral diversity in areas of high population density (30). We also noted a significantly higher rate of viral positivity on multiplex PCR among the London participants compared with the Groningen participants (22 of 144 [15.3%] Groningen participants; 126 of 257 [49.0%] in London).

In terms of limitations, it should be noted that the focus of the present study was on RSV detection, with less emphasis on diagnosing other viruses such as rhinovirus. However, we have shown that the diagnostic method was not related to the severity of infection (Figure 2B). The completion of sample collection before severe acute respiratory syndrome coronavirus 2 (SARS-CoV-2) became widespread in the United Kingdom and the Netherlands is arguably a major study strength, with respect to understanding the role of RSV outside of a pandemic period. Public health measures used to mitigate viral transmission during the pandemic substantially impacted RSV infections, seasonality, and RSV genetic diversity (31). Although RSV infection patterns appear to be returning to prepandemic patterns (32), the long-term effects of coronavirus disease (COVID-19)’s direct and indirect disruption of seasonal epidemiological patterns remain to be determined (32).

Our study focused on adult RSV infection in the community, providing valuable RSV data from this less-studied care setting. However, during the study period, 15 participants died and 14 participants in London required hospital admission. RSV infections associated with these terminal or hospitalization events may therefore have been missed. None of the deaths were related to exacerbations studied in the present work. Designing studies that span the community and hospital settings remains challenging, potentially leading to an underestimation of the role of RSV in community settings.

In conclusion, RSV accounted for approximately 8.7% of all mild to moderate COPD exacerbations in community COPD cohorts. RSV-related exacerbations appear to be of shorter duration than RSV-negative exacerbations, and lower airway sampling (i.e., sputum) achieved a higher rate of RSV detection than upper airway sampling (i.e., NP swabs) in patients with COPD. We suggest that an increase in RSV N-specific antibody may be a useful additional diagnostic modality in the retrospective identification of RSV disease in longitudinal studies, even after the rollout of vaccines aimed at the prevention of RSV disease.

## Supplemental Materials

10.1164/rccm.202308-1320OC ONLINE DATA SUPPLEMENT
