# Supplementary material for: Respiratory Syncytial Virus–related Community Chronic Obstructive Pulmonary Disease Exacerbations and Novel Diagnostics: A Binational Prospective Cohort Study
Source: Am J Respir Crit Care Med. 2024 Mar 19;210(8):994–1001. doi: 10.1164/rccm.202308-1320OC (PMC11531101; doi:10.1164/rccm.202308-1320OC)
Supplement: ONLINE DATA SUPPLEMENT [file rccm.202308-1320OCS1.docx]

**RSV-related Community COPD Exacerbations and Novel Diagnostics: A Binational Prospective Cohort Study**

**ONLINE DATA SUPPLEMENT**

**Table of Contents:**

Table E1: Causes of Exacerbation Categorization 2

Bacteriology associated with exacerbations 2

Figure E1 – Graph showing PCR positivity from NP swabs at exacerbation 3

Figure E2 - Sputum exacerbation samples 3

Figure E3 - Sputum Microbiology Results at Exacerbation – London 3

Table E2 - Time to recovery (days) in RSV-associated exacerbations vs other exacerbation causes 3

Figure E4 - FEV1 (%) Change during exacerbation 3

Change in CAT Score 4

Recruitment Methods and Success Rates 4

Figure E5 - Overall Recruitment Success Rates by Method 5

Figure E6 – Correlation of Serological Analysis between ELISA and pentaplex 5

Table E3 - Annual Review Form 6

References 9

**Table E1: Causes of Exacerbation Categorization:**

- Unknown: which included all exacerbations where no cause was found by analysis of sputum or NP swabs, where no sputum or NP swab was analysed, or where only colonizing bacteria were found in the sputum.
- RSV-associated: where RSV was found on NP swab PCR, sputum PCR or RSV was positive through serology
- Rhinovirus-associated: where rhinovirus was found on NP swab PCR or sputum PCR.
- Flu associated: where Flu (Influenza (Flu) A, Flu B, Flu A (California/7/2009 H1N1), or Flu A (H3)) were found on NP swab PCR or sputum PCR.
- Other Viral: where other viruses (human coronavirus (HCV)-NL63, HCV-229E, HCV-OC43, human bocavirus, human enterovirus, human adenovirus, parainfluenza virus (PIV)-1, PIV-2, PIV-3, PIV-4, or human metapneumovirus (HMV)) were found on NP swab PCR or sputum PCR.
- Bacteria-associated: where non-colonising bacteria were found on sputum microbiology at the Royal Brompton microbiology laboratory. Any colonising bacteria previously seen at baseline visits for the participant, not previously causing an exacerbation were excluded.
- Viral + Bacterial: Where viral and bacterial pathogens were discovered during the same exacerbation.

**Bacteriology associated with exacerbations**

Full PCR results for NP swabs and sputum are shown in supplementary Figures E1 + E2 respectively. In the London cohort sputum was sent per local protocol to the Royal Brompton hospital laboratories for bacterial analysis of sputum culture (Figure E3). These results were used to guide clinical decisions such as antibiotic treatment. The most common causes were Moraxella, Pseudomonas, Staphylococcus aureus, Haemophilus influenzae and Streptococcus pneumoniae.

**Full PCR Results – London + Groningen**

Figure E1 - Graph of NP swabs at exacerbation reaching threshold for positivity using qPCR. Groningen in orange, London in blue. London (N=57) Groningen (N=13).

Figure E2 - Sputum exacerbation samples reaching threshold for positivity using qPCR according to site. London (N=67) Groningen (N=9).

Figure E3 – Sputum Microbiology Results at Exacerbation – London (N=77)

Table E2 – Kruskal-Wallis and Dunn’s test for time to recovery (days) in RSV-associated exacerbations vs other exacerbation causes.

| RSV-related vs Unknown | *P=0.*0131 using Dunn’s Test |
| --- | --- |
| RSV-related vs Rhinovirus-related | *P=0.*0003 using Dunn’s Test |
| RSV-related vs Flu-related | *P=0.*0259 using Dunn’s Test |
| RSV-related vs other virus-related | *P=0.*0157 using Dunn’s Test |
| RSV-related vs Bacteria-related | *P=0.*0320 using Dunn’s Test |
| RSV-related vs Bacterial and Viral Co-Infection | *P=0.*0210 using Dunn’s Test |

**FEV1 Percentage Change**

Exacerbations associated with rhinovirus (n=30), other respiratory viruses (n=16), bacterial infection (n=24), or unknown cause (n=64) were all associated with significant decreases in FEV1 during exacerbations. RSV-associated exacerbations and Flu-associated exacerbations had a trend towards decreased FEV1 but did not meet statistical significance (P=0.065). The lack of significance could be due to low numbers and a lack of statistical power (Table 2).

Figure E4 - FEV1 (%) Change during exacerbation, by exacerbation causes.

**Change in CAT Score**

Change in CAT score (relative to preceding baseline visit) for each exacerbation-associated pathogen group was also analysed. There was a trend towards higher scores (representing greater severity) in RSV- and Flu-related exacerbations, but this was not statistically significant. There was a statistically significant difference between CAT scores of RSV associated exacerbations and CAT scores of exacerbations associated with the ‘other viruses’ group, with RSV-associated demonstrating the greater change (*P=*0.019). There was also a statistically significant difference between the CAT score in Flu associated exacerbations and those associated to the ‘other viruses’ group (*P=*0.016) (Table 2).

**Recruitment Methods and Success Rates:**

**The London COPD Cohort**

The London COPD Exacerbation Cohort (EXCEL Cohort) is an established cohort designed to study mechanisms of COPD exacerbations and their impact on health status and disease progression. Our work has contributed greatly to the understanding of COPD exacerbations. The cohort has already participated in observational studies, proof of concept studies, both grant-funded and industry-funded, to reduce the frequency and severity of these important events. We will utilize our existing strengths for accurately determining exacerbation frequency and severity with robust methodology previously developed by our group. Patients will be trained to monitor their symptoms electronically and detect and report exacerbations promptly, so that their exacerbations can be sampled early after symptom onset.

**Advertisement**

The first method was placing an advertisement in the local newspaper (Kensington and Chelsea Times) (1). The advertisement was written by our study team and had both ethical approval (as discussed in the methods), and approval by The Royal Brompton Foundation Trust prior to publication. Out of 15 individuals who contacted us by telephone, only three met the telephone screening criteria (as per methods) and were invited to a screening visit. Of those three potential participants, only one met the inclusion criteria and was eventually recruited into the study as a participant (1/15 6.67%). We did not try any other local newspapers, as the recruitment from this method was so poor on the initial attempt. Despite the circulation advertised as being ~200,000 and the paper being out every three months, there was little interest shown for this advertisement.

**Discover Register**

The second method utilised the newly created ‘Discover’ register (2). This is a private register of patients in North-West London who have agreed to be contacted for medical research purposes. They have each been coded (by the system) with any medical conditions or diagnoses they have. We asked to see a list of those coded as COPD. Of the 38 individuals contacted, 18 passed the telephone screening and were sent a copy of the participant information sheet (PIS). Out of those 18 individuals, nine attended for a recruitment visit and of those, three participants were recruited into the study.

**West London Lung Cancer Screening Study**

The third method of recruitment used was to contact participants enrolled in the West London Lung Cancer Screening Study (3). These patients were pre-screened for respiratory obstruction, with spirometry in the community, before entering the cancer screening study. They had also signed a form consenting to contact for recruitment to other studies. From an initial list of 195 patients, 169 participants were electronically screened based on electronic patient records (EPR). Of those 169, 111 appeared to meet the inclusion criteria and were contacted. Of those 111 contacted, 47 showed an interest and were sent a PIS. This resulted in 28 individuals being booked in for a screening visit. Out of those 28 individuals, 26 met the inclusion criteria and were recruited into the study as participants.

**London EXCEL COPD Cohort**

The majority of participants were recruited directly from London EXCEL COPD Cohort(4). The inclusion/exclusion criteria for the COPD RSV study (this study) were created with the EXCEL COPD Cohort in mind and, therefore, identical. This means that all of the patients in the EXCEL COPD Cohort should theoretically meet the inclusion criteria for the COPD RSV study. Out of the 149 individuals contacted and sent the PIS, 147 agreed to join and were recruited into the COPD RSV study as participants.

Figure E5 – Overall Recruitment Success Rates by Method

Figure E6 – Correlation between IgG titres against A) Pre-F and B) Post-F measurements between the ELISA and Pentaplex methods (data log10 transformed). X-axes display ELISA data, Y-axes display Pentaplex data per antigen. Analysis of correlations were tested by Pearson’s test.

Table E3 – Annual Review Form

**Annual review (V1) THE LONDON COPD COHORT/RESCEU**

| **Dyspnoea and wheeze** | | | | | | | | | | | |
| --- | --- | --- | --- | --- | --- | --- | --- | --- | --- | --- | --- |
| 1. When are you short of breath? (please tick) | | | | | | | | | | | |
| All day |  | Only in the mornings | | |  | 1-2x a week | | | | |  |
| Only on exercise |  | Only with chest infections | | |  | Never | | | | |  |
| 1. When do you wheeze? (please tick) | | | | | | | | | | | |
| All day |  | Only in the mornings | | |  | 1-2x a week | | | | |  |
| Only on exercise |  | Only with chest infections | | |  | Never | | | | |  |
| **Chronic bronchitis and cough** | | | | | | | | | | | |
| 1. Does your cough wake you up at night? | | | | | | Yes |  | | No | |  |
| 1. Do you bring up phlegm (sputum) for more than 3 months during the year? | | | | | | Yes |  | | No | |  |
| 1. On average how much sputum do you bring up in a day? | | | | | | | | | | | |
| None |  | < egg cup (<30 mL) | | |  | >egg cup (>30 mL) | | | | |  |
| 1. On an average day what is the colour of your sputum? | | | | | | | | | | | |
| White |  | Yellow | | |  | Green | | | | |  |
| Brown |  |  | | |  |  | | | | |  |
| **Exacerbations** | | | | | | | | | | | |
| 1. How many exacerbations have you had over the last 12 months? | | | | | | | | | | | |
| Total exacerbations |  | Treated exacerbations | | |  | Hospital admissions | | | | |  |
| Steroid courses |  | Antibiotic courses | | |  | Bone protection | | | | |  |
| **Smoking** | | | | | | | | | | | |
| 1. Do you currently smoke? | | | | | | Yes |  | | No | |  |
| 1. Are you an intermittent quitter? | | | | | | Yes |  | | No | |  |
| Age started smoking |  | Age quit smoking | | |  | Cigarettes/rollups/day | | | | |  |
| 1. If still smoking what/how may are you smoking (number/day for all that apply) | | | | | | | | | | | |
| Cigarettes |  | Roll ups | | |  | e-cigarettes | | | | |  |
| 1. If still smoking would you like to quit? | | | | | | Yes | |  | | No |  |
| 1. Advice/information offered? | | | | | | Yes | |  | | No |  |
| **Atopy** | | | | | | | | | | | |
| 1. History of atopy – please tick all that apply | | | | | | | | | | | |
| Do you have asthma? |  | Do you have hay fever? | | |  | Do you have eczema? | | | | |  |
| Have you had skin prick tests? | | Yes |  | No |  | Results | | | | | |
| **Other medical problems** | | | | | | | | | | | |
| **Pulmonary rehabilitation** | | | | | | | | | | | |
| 1. Have you completed a pulmonary rehabilitation course? | | | | | | Yes | |  | | No |  |
| Date started |  | Date finished | | |  | Still exercising? | | | | |  |
| 1. **Immunisation** | | | | | | | | | | | |
| Have you had a flu vaccine? | | Yes |  | No |  | Dates | | | | |  |
| Pneumonia vaccine? | | Yes |  | No |  | Dates | | | | |  |
| 1. **Nasal symptoms** | | | | | | | | | | | |
| Nasal symptoms over 3 months (0= no symptoms, 1= v.mild, 2=mild, 3=mod, 4=severe, 5=bad as can be) | | | | | | | | | | | |
| Runny |  | PND | | |  | Blocked | | | | |  |
| Sneezing |  | Anosmia | | |  | Sum | | | | |  |
| 1. **Medication history** | | | | | | | | | | | |
| Medication | Dose | Units | Frequency | Route | Indication | | | | | Start | Stop |
|  |  |  |  |  |  | | | | |  |  |
|  |  |  |  |  |  | | | | |  |  |
| Allergies |  | | | | | | | | | | |
| **Social History** | | | | | | | | | | | |
| 1. Marital status | | | | | | | | | | | |
| Married/civil partner |  | Widow/ed | | |  | Divorced/separated | | | | |  |
| Single |  | Other | | |  |  | | | | |  |
| 1. Number of children | |  | | | Age of youngest child | | | | | |  |
| 1. Number of times out of home/week | | | |  | Number of visitors to home/week | | | | | |  |
| Times contact with children < 16 years last week | | >2 |  | 1-2 |  | <1 | |  | | none |  |
| 1. **Accommodation and pets** | | | | | | | | | | | |
| House |  | Flat | | |  | Maisonette | | | | |  |
| Bungalow |  | Other | | |  |  | | | | |  |
| Number of occupants in home | | | | | | | | | | | |
| Partner/spouse |  | Children | | |  | Siblings | | | | |  |
| Parents |  | Others | | |  | Lives alone | | | | |  |
| Family pets | | | | | | | | | | | |
| Cats |  | Dogs | | |  | Birds | | | | |  |
| None |  | Others | | |  | | | | | | |
| 1. **Clinical findings** | | | | | | | | | | | |
| SaO2 |  | FiO2 | | |  | Heart rate bpm | | | | |  |
| BP mmHg |  | Steroid bruising | | |  | Peripheral oedema | | | | |  |
| Finger clubbing |  | Height cm | | |  | Weight kg | | | | |  |
| **Spirometry** | | | | | | | | | | | |
| FEV1 |  | |  | |  | | | % predicted | | | |
| FVC |  | |  | |  | | | % predicted | | | |
| **Questionnaires** | | | | | | | | | | | |
| SGRQ |  | Depression scale | | |  | Fatigue score | | | | |  |
| MRC scale |  | VAS | | |  | CAT | | | | |  |
| **Bioimpedance** | | | | | | | | | | | |
| %Fat |  | Fat kg | | |  | %FFM | | | | |  |
| FFM kg |  | Total body water | | |  |  | | | | |  |
| Basal metabolic rate |  | BMI | | |  | Impedance | | | | |  |
| **Other investigations (tick completed)** | | | | | | | | | | | |
| ECG |  | Sputum | | |  | Urine | | | | |  |
| Blood + **1 extra red top** |  | Book lung function | | |  | **NP swab** | | | | |  |

References:

1. Kensington and Chelsea Times. at <http://www.kctimes.co.uk/index.html>.

2. Home | Discover. at <https://www.registerfordiscover.org.uk/>.

3. Bartlett EC, Kemp S V, Ridge CA, Desai SR, Mirsadraee S, Morjaria JB, *et al.* Baseline Results of the West London lung cancer screening pilot study – Impact of mobile scanners and dual risk model utilisation. 2020;doi:10.1016/j.lungcan.2020.07.027.

4. The London COPD Exacerbation Cohort - Full Text View - ClinicalTrials.gov. at <https://clinicaltrials.gov/ct2/show/NCT02755974>.
